# Non-canonical peroxisome targeting signals: identification of novel PTS1 tripeptides and characterization of enhancer elements by computational permutation analysis

**DOI:** 10.1186/1471-2229-12-142

**Published:** 2012-08-11

**Authors:** Gopal Chowdhary, Amr RA Kataya, Thomas Lingner, Sigrun Reumann

**Affiliations:** 1 Centre for Organelle Research, University of Stavanger, N-4036, Stavanger, Norway; 2 KIIT School of Biotechnology, Campus XI, KIIT University, Bhubaneswar, 751024, India; 3 Department of Bioinformatics, Institute for Microbiology and Genetics, D-37077, Goettingen, Germany

## Abstract

**Background:**

High-accuracy prediction tools are essential in the post-genomic era to define organellar proteomes in their full complexity. We recently applied a discriminative machine learning approach to predict plant proteins carrying peroxisome targeting signals (PTS) type 1 from genome sequences. For *Arabidopsis thaliana* 392 gene models were predicted to be peroxisome-targeted. The predictions were extensively tested *in vivo*, resulting in a high experimental verification rate of Arabidopsis proteins previously not known to be peroxisomal.

**Results:**

In this study, we experimentally validated the predictions in greater depth by focusing on the most challenging Arabidopsis proteins with unknown non-canonical PTS1 tripeptides and prediction scores close to the threshold. By *in vivo* subcellular targeting analysis, three novel PTS1 tripeptides (QRL>, SQM>, and SDL>) and two novel tripeptide residues (Q at position −3 and D at pos. -2) were identified. To understand why, among many Arabidopsis proteins carrying the same C-terminal tripeptides, these proteins were specifically predicted as peroxisomal, the residues upstream of the PTS1 tripeptide were computationally permuted and the changes in prediction scores were analyzed. The newly identified Arabidopsis proteins were found to contain four to five amino acid residues of high predicted targeting enhancing properties at position −4 to −12 in front of the non-canonical PTS1 tripeptide. The identity of the predicted targeting enhancing residues was unexpectedly diverse, comprising besides basic residues also proline, hydroxylated (Ser, Thr), hydrophobic (Ala, Val), and even acidic residues.

**Conclusions:**

Our computational and experimental analyses demonstrate that the plant PTS1 tripeptide motif is more diverse than previously thought, including an increasing number of non-canonical sequences and allowed residues. Specific targeting enhancing elements can be predicted for particular sequences of interest and are far more diverse in amino acid composition and positioning than previously assumed. Machine learning methods become indispensable to predict which specific proteins, among numerous candidate proteins carrying the same non-canonical PTS1 tripeptide, contain sufficient enhancer elements in terms of number, positioning and total strength to cause peroxisome targeting.

## Background

Revealing the subcellular localization of unknown proteins is of major importance for inferring protein function. Major progress has been made in the past few years in experimental proteomics technology. As a result, many novel organellar proteins have been identified and their physiological functions have been defined at the molecular level. Despite this success, these experimental methods are limited in protein identification capabilities by several parameters, for instance, by technological sensitivity and organelle purity, and to major plant tissues and organs. This holds true particularly for small and fragile organelles such as peroxisomes that can only be isolated in sufficient purity and quantity from a few plant species, generally only from one tissue type per organism (leaves, cotyledons, or endosperm) and only from organisms raised under optimal growth conditions. As a result, most low-abundance proteins of peroxisomes have remained unidentified to date. Complementary to experimental proteome research, protein targeting prediction from genome sequences has emerged as a central and essential tool in the post-genomic era to define organellar proteomes and to understand metabolic and regulatory networks [1-4].

Peroxisomes are small, ubiquitous eukaryotic organelles that mediate a wide range of oxidative metabolic activities. Classical physiological functions of plant peroxisomes are lipid metabolism, photorespiration, and hormone biosynthesis (e.g., jasmonic acid and indole acetic acid; [5-7]). Additionally, many novel plant peroxisome functions, for instance in secondary metabolism, have been uncovered in the past few years (for review, see [8,9]). Agronomically most important, plant peroxisomes also play pivotal roles in responses to and tolerance of abiotic and biotic stresses [10,11].

Soluble matrix proteins of peroxisomes are imported directly from the cytosol [12]. Apart from a few exceptions, proteins are targeted to the peroxisome matrix by a conserved peroxisome targeting signal of either type 1 (PTS1) or type 2 (PTS2). The motifs of both targeting peptides have been applied to predict and identify matrix proteins from genome sequences [13-15]. Computational prediction methods have been developed for PTS1/2 proteins in animals and fungi, but have long been missing specifically for plants. Such plant-specific tools are needed because plant peroxisomal proteins with non-canonical PTS1 tripeptides can, in general, not be predicted correctly by algorithms developed for metazoa [16]. Non-canonical PTS1 tripeptides (e.g., SSI>, ASL>, and SLM > for plants; “>” indicates the C-terminal end of the protein; [17,18]) are generally restricted to a few, preferentially low-abundance (weakly expressed), peroxisomal proteins. These tripeptides alone generally represent weak signals that require auxiliary targeting-enhancing patterns (e.g., basic residues) for functionality, which are located immediately upstream of the tripeptide and are often kingdom specific.

To increase the number of known plant PTS1 proteins, we developed proteomic methods for *Arabidopsis* leaf peroxisomes and identified more than 90 putative novel peroxisomal proteins, including many long-awaited low-abundance and regulatory proteins [17,18]. By *in vivo* targeting analysis and PTS identification, many novel *Arabidopsis* PTS1 proteins have been established in the past few years (http://www.peroxisome.msu.edu; [17-23]; for review see [8,9]). These experimental data provided a solid foundation for the development of PTS1 protein prediction methods.

Using 60 known *Arabidopsis* PTS1 proteins as queries for homology searches, we generated a large dataset of more than 2,500 homologous sequences that derived from approx. 260 different plant species and primarily from EST databases. Upon application of a discriminative machine learning approach, two different prediction methods were derived, both of which showed high prediction accuracy for diverse plant species and recognized specific targeting-enhancing patterns in the regions upstream of the PTS1 tripeptides [16]. Both prediction methods showed high accuracy on example sequences of diverse plant species and were able to correctly infer novel PTS1 tripeptides, even including novel residues. In combination with large-scale *in vivo* subcellular targeting analyses, the prediction methods were proven to be suitable for the prediction of plant peroxisomal PTS1 proteins from genomic sequences, as demonstrated representatively for Arabidopsis, including low-abundance and non-canonical PTS1 proteins [16].

Nevertheless, one major challenge remains, namely to correctly predict plant proteins carrying non-canonical PTS1 tripeptides. First, many non-canonical PTS1 tripeptides have remained unidentified to date. Second, among a large number of plant proteins carrying one and the same non-canonical PTS1 tripeptide, only a small subset is indeed peroxisome-targeted. The reason is that non-canonical PTS1 tripeptides alone are generally weak, and require auxiliary targeting-enhancing patterns (e.g., basic residues) located immediately upstream of the tripeptide, to target proteins to peroxisomes. Knowledge about the identity and positioning of these enhancer patterns is lacking, making correct peroxisome targeting predictions challenging. By contrast, canonical PTS1 tripeptides are stand-alone targeting signals that generally do not require enhancer elements. The predominance of canonical PTS1 tripeptides such as SKL > (26.7 %, 655 out of 2562 sequences) among positive example sequences [16] makes the recognition of specific discriminative features in the relatively low number of known non-canonical PTS1 proteins difficult.

In this study, we validated the algorithms in greater depth by focusing on the most challenging predictions, namely Arabidopsis proteins (i) with PTS1 protein prediction scores close to the prediction threshold, (ii) containing non-canonical, yet unknown PTS1 tripeptides, and (iii) carrying putatively novel residues in the PTS1 tripeptide. By *in vivo* subcellular targeting analysis, three novel plant PTS1 tripeptides and two novel tripeptide residues were identified, further extending the relaxation degree of the plant PTS1 motif at single positions. Our newly established single-residue computational permutation analysis of specific non-canonical Arabidopsis proteins of interest identifies particular amino acid residues in the upstream domain that are predicted to strongly enhance peroxisome targeting.

## Results

### Selection of predicted Arabidopsis PTS1 proteins for experimental validation

To validate the algorithms in greater depth, we selected further Arabidopsis proteins of interest that followed specific criteria. First, we chose proteins that had been assigned PTS1 protein prediction scores close to the threshold. The PTS1 protein prediction scores of the five Arabidopsis proteins spanned the range of 0.336 to 0.450 with the threshold for peroxisome targeting located at 0.412 (Table1, [16]). Second, we selected Arabidopsis proteins containing yet unknown non-canonical PTS1 tripeptides (HKL>, RKM>, SQM>, QRL>, and SDL>). Third, we focused on proteins that preferentially carried putative novel PTS1 tripeptide residues, i.e. either at position −3 (H, R, and Q in HKL>, RKM>, QRL>, potentially novel residues underlined) or at position −2 (D in SDL>, Table1). None of these potentially novel PTS1 tripeptide residues had been described previously as an allowed residue in plant PTS1s ([14,16,24,25]). Fourth, we prioritized Arabidopsis proteins (e.g., heat-shock proteins, HSP70T-2, At2g32120; DNAJ homolog, At1g18700, Table1) whose localization in the peroxisome matrix had long been postulated but not yet been demonstrated. Except for the protein terminating with SDL>, all Arabidopsis proteins were predicted as peroxisome-targeted by the PWM prediction model, while the scores given by the alternative and more stringent RI model were generally below threshold [16].

**Table 1:** Summary of prediction data for selected Arabidopsis proteins carrying novel PTS1 tripeptides and/or residues

**AGI code**	**Annotation**	**Acronym**	**C-terminal 14 aa**	**PWM model**								**RI model**			***In vivo*subcelluar targeting**	**Figure**2
				**Hier. pos.**	**Pred. score**	**Post prob.**	**Perox. pred.**	**Hier. pos.**	**Pred. score**	**Perox. pred.**						
At5g50580.1/2/At5g50680.1/2	SUMO-activating enzyme 1B	SAE1B	EDGKGVIEDLSH**KL**>	315^1^	0,450	0,719	P	473^2^	0,013	C	cytosol	B
At2g32120.1/2	Heat-shock protein 70 T-2	HSP70T-2	YGATLDLITLQR**KM**>	321^3^	0,448	0,706	P	1100	0,207	C	cytosol	C
At5g45160.1	ROOT HAIR DEFECTIVE 3 homolog 2	RHD3H2	RNTNNVQESEI**SQM**>	337	0,440	0,667	P	619	0,073	C	peroxisome	E, F, J
At1g18700.2	DNAJ heat shock N-terminal domain-containing protein	DNAJ	ILSSVRSMKGFQ**RL**>	338	0,440	0,666	P	300	0,082	C	peroxisome	D, I
At5g03730.1/2	CONSTITUTIVE TRIPLE RESPONSE 1 (protein kinase superfamily protein)	CTR1, SIS1	LIKSAVPPPNR**S**D**L**>	522/3	0,336	0,117	C	632/3	0,079	C	peroxisome	G, H, K

The three alternative gene models for the DNAJ heat-shock protein (At1g18700.1/3/4) differ in the C-terminal domain (Figure3) and are not predicted as peroxisome-targeted by the PTS1 pathway (PWM prediction pos. 30486–8, PWM score −1.298). Potentially novel PTS1 tripeptide residues are underlined. ‚^1^ 315–18, ^2^ 473–476, ^3^ 321/2.

### Arabidopsis proteins with novel residues at PTS1 tripeptide position −3

**Figure 1:**
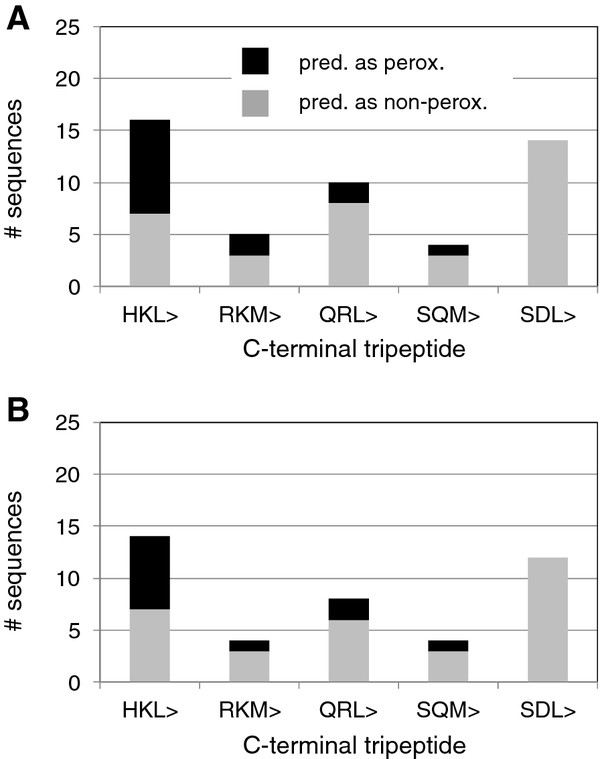
**Number of Arabidopsis gene models (A) and gene loci (B) terminating with one of five predicted non-canonical PTS1 tripeptides.** The number of Arabidopsis gene models (loci) predicted as peroxisomal is indicated by black columns, and those predicted as non-peroxisomal are represented by grey columns.

We first studied Arabidopsis proteins with potentially novel PTS1 tripeptide residues at pos. -3. Three putatively novel non-canonical PTS1 tripeptides, namely HKL>, QRL>, and RKM>, were chosen. Neither His (H), Gln (Q), nor Arg (R) had been previously validated as functional PTS1 tripeptide residues at pos. -3. Among 16 Arabidopsis gene models (14 gene loci) terminating with HKL>, nine were predicted peroxisomal by the PWM prediction model (Figure1, [16]). The Arabidopsis HKL > decapeptide chosen for experimental validation derived from SUMO-activating enzyme 1B (At5g50580.1). The PWM model prediction score of 0.450 was slightly above the prediction threshold (0.412), as indicated by a posterior probability of 0.719 (0.5 at threshold, Table1).

**Figure 2:**
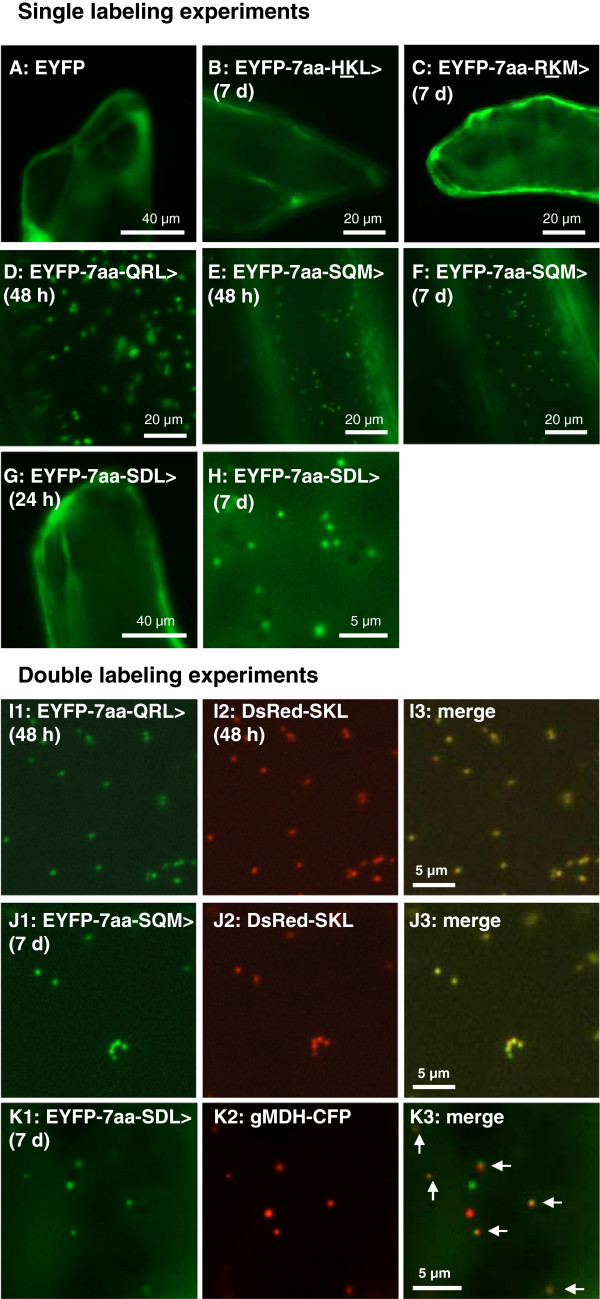
**Experimental validation of Arabidopsis proteins carrying newly predicted non-canonical PTS1 domains by*in vivo*subcellular targeting analysis.** Onion epidermal cells were transformed biolistically with EYFP fusion constructs that were C-terminally extended by the C-terminal decapeptides of Arabidopsis proteins carrying newly predicted non-canonical PTS1 domains. Subcellular targeting was analyzed by fluorescence microscopy after ca. 48 h expression (ca. 18 h RT plus 30 h ca. 10 °C), or ca. 7 d (ca. 18 h RT plus 6 d ca. 10 °C). Cytosolic constructs, for which subcellular targeting data are shown after short-term expression times, were reproducibly confirmed as cytosolic also after long-term expression. Possibly novel aa residues of PTS1 tripeptides are underlined. To document the efficiency of peroxisome targeting, EYFP images of single transformants were not modified for brightness or contrast (A-H). In double transformants, peroxisomes were labeled with DsRed-SKL or PTS2-CFP with cyan fluorescence having been converted to red for image overlay (I-K). In Figure2K the arrows point at six EYFP-labeled peroxisomes (yellow), while two organelles are only fluorescing in red or green, most likely due to quick organelle movement. EYFP alone was included as negative control (A).

To experimentally investigate whether the Arabidopsis protein indeed contained a functional PTS1 domain, we extended the reporter protein enhanced yellow fluorescent protein (EYFP) C-terminally by the predicted PTS1 domain comprising the C-terminal ten amino acid (aa) residues of the Arabidopsis proteins. This method had been successfully established previously [16,17,19,26-28]. Compared to full-length protein fusions, this approach has the advantage that possible negative effects of polypeptide conformation are reduced to a minimum. The construct was transiently expressed from the 35 S cauliflower mosaic virus (CaMV) promoter in onion epidermal cells. Plant tissue transformed with a negative control plasmid, such as EYFP alone (Figure2A), showed uniformly cytosolic and nuclear fluorescence. The reporter construct EYFP-7aa-HKL > was cytosolic/nuclear under standard conditions of gene expression (18 h RT, data not shown). We had previously observed that extended incubation times at reduced temperature increased the sensitivity in detecting peroxisome targeting for several (but not all) reporter protein constructs [16]. When this methodology was applied to EYFP-7aa-HKL>, the construct remained undetectable in punctuate subcellular structures (Figure2B).

Among five Arabidopsis gene models (four gene loci) terminating with RKM>, two were predicted peroxisomal by the PWM prediction model (Figure1). The Arabidopsis RKM > decapeptide chosen for experimental validation derived from heat-shock protein 70 T-2 (At2g32120), which had been assigned the PWM prediction score of 0.448 (posterior probability 0.706, Table1). In experimental subcellular targeting studies the reporter construct EYFP-7aa-RKM > was cytosolic/nuclear under both standard and extended conditions of gene expression (Figure2C).

**Figure 3:**
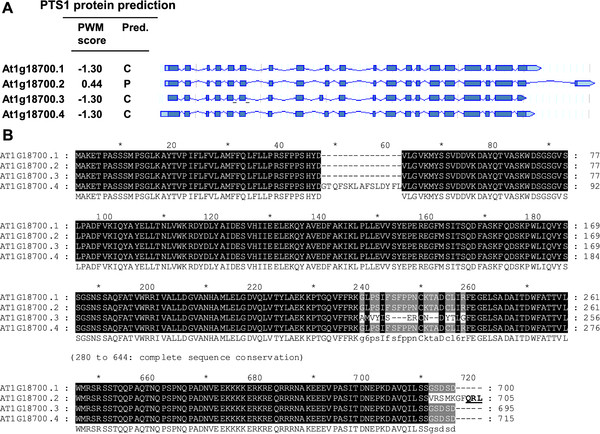
**Transcriptional variants of a DNAJ homolog (At1g18700) with one splice variant carrying the PTS1 tripeptide QRL > and a functional PTS1 domain.** (**A**) Schematic diagram showing the CDS structure and multiple sequence alignment and (**B**) multiple sequence alignment of four transcriptional variants (At1g18700.1-4) of an Arabidopsis DNAJ homolog.

**Figure 4:**
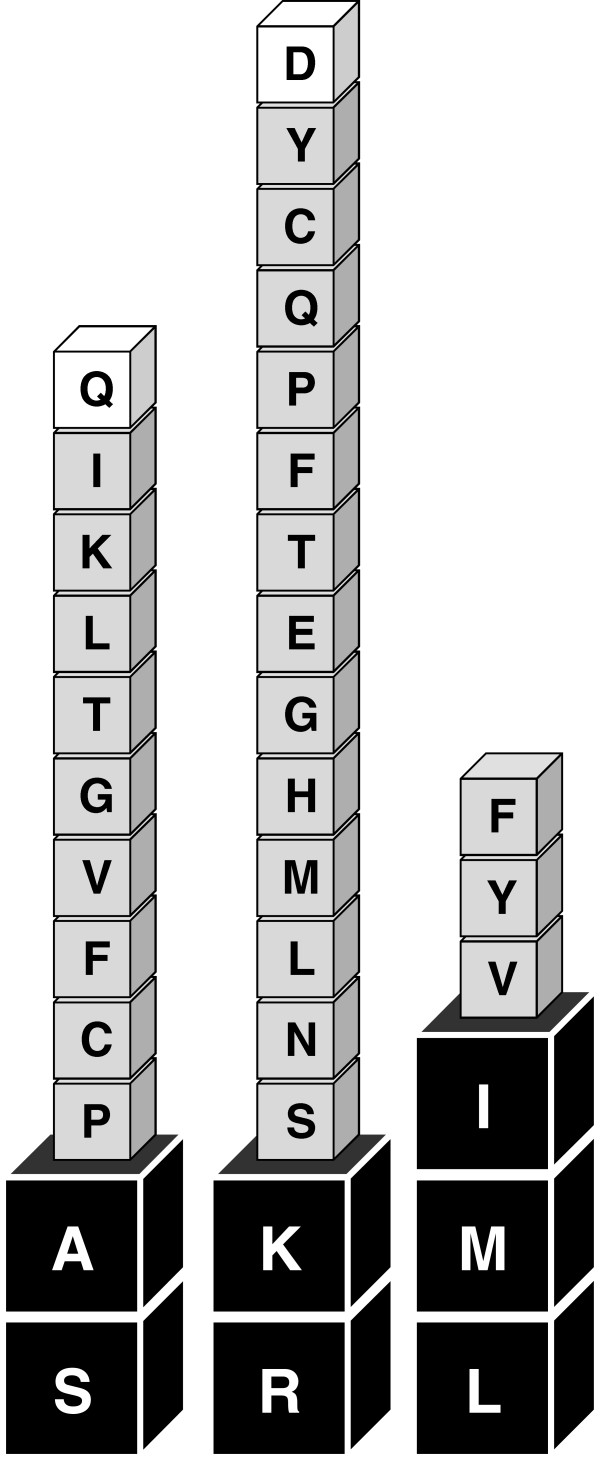
**Experimentally validated aa residues of the plant PTS1 motif.** Tripeptide residues identified as novel plant PTS1 tripeptides in this study (Q at pos. -3 and D at pos. -2) are indicated by white boxes. According to experimental data and PWM model predictions [16], at least two of the seven high-abundance residues of predicted high targeting strength ([SA][KR][LMI]>, black boxes) must be combined with one low-abundance residue (grey or white boxes) to yield functional plant PTS1 tripeptides (x[KR][LMI]>, [SA]y[LMI]>, [SA][KR]z>).

Among ten Arabidopsis gene models (eight gene loci) terminating with QRL>, two were predicted peroxisomal by the PWM prediction model (Figure1). The Arabidopsis QRL > decapeptide chosen for experimental validation derived from a DNAJ homolog (At1g18700). Among in total four transcriptional variants, variant 2 (At1g18700.2) specifically terminated with QRL > (Figure3A, B) and was assigned the prediction score 0.440, while the other three variants were among those 5,000 (out of 35,386) Arabidopsis gene models with the lowest scores (PWM score −1.30; At PTS1 protein prediction pos. 30486–8). The reporter protein EYFP-7aa-QRL > targeted small punctuate structures with low cytosolic background fluorescence under standard conditions of gene expression (48 h RT, Figure2D), indicating a relatively high peroxisome targeting strength of the decapeptide. The identity of the punctuate structures with peroxisomes was confirmed in double transformants co-expressing the peroxisome marker *DsRed-SKL* (Figure2I). The results demonstrated that (i) QRL > is a novel functional PTS1 tripeptide, (ii) Q is a novel PTS1 tripeptide residue at pos. -3 (Figure4), and that (iii) the second splice variant of the DNAJ homolog At1g18700 carries a functional PTS1 domain.

### Arabidopsis proteins with novel residues at position −2 of the PTS1 tripeptide

We next investigated constructs with potentially new residues at pos. -2, which had long been assigned a requirement for basic residues. We had previously verified by experimental analyses that a total of 15 aa residues are allowed at pos. -2 in plant PTS1 proteins. Apart from basic residues (R, K, H) the compatible residues included neutral residues (e.g., leucine, L, and glycine, G) and even the acidic residue, glutamate (E, [16]). Two putatively novel non-canonical PTS1 tripeptides, namely SQM > and SDL>, were chosen. Among four Arabidopsis gene models (four gene loci) terminating with SQM>, one was predicted peroxisomal by the PWM prediction model (Figure1, Table1). The Arabidopsis SQM > decapeptide chosen for experimental validation derived from ROOT HAIR DEFECTIVE 3 (RHD3) homolog 2 (RHD3H2, At5g45160.1). The PWM model prediction score of 0.440 was close to the prediction threshold (0.412), consistent with a posterior probability of 0.667. The reporter protein construct extended by the predicted PTS1 domains terminating with SQM > remained cytosolic under standard conditions of gene expression (data not shown). However, after slightly extended times of expression (48 h), small yellow fluorescent punctuate structures became visible that moved quickly along cytoplasmic strands, indicating that the reporter construct targeted peroxisomes with weak efficiency (Figure2E). Extended expression times up to seven days at ca. 10°C improved visualization of peroxisome targeting in a few, but not all cells (Figure2F). The identity of the fluorescent organelles with peroxisomes was confirmed in double transformants co-expressing *DsRed-SKL* (Figure2J). The data validated SQM > as a novel plant PTS1 tripeptide.

Among 14 Arabidopsis gene models (twelve gene loci) terminating with SDL>, none were predicted peroxisomal by the PWM prediction model (Figure1, Table1). The Arabidopsis SDL > decapeptide chosen for experimental validation was derived from CONSTITUTIVE TRIPLE RESPONSE 1 (CTR1/SIS, At5g03730.1/2). The PWM model prediction score of 0.336 was below the prediction threshold (0.412) and had been assigned a low posterior probability of 0.117. CTR1/SIS had been assigned the third highest PWM prediction score among the twelve Arabidopsis gene models terminating with SDL > [16].

The reporter construct EYFP-7aa-SDL > with the atypical acidic residue Asp at pos. -2 remained cytosolic under short-term conditions of gene expression but could be detected in small punctuate structures after long-term expressions (Figure2G, H). The punctuate structures were shown to coincide with peroxisomes (Figure2K), thereby validating SDL > as a novel plant PTS1 tripeptide and Asp as a novel plant PTS1 tripeptide residue at pos. -2 (Figure4).

In summary, the experimental analyses demonstrated that (i) three tripeptides are novel functional plant PTS1 tripeptides (QRL>, SQM>, and SDL>), (ii) Q and D are novel PTS1 tripeptide residues at pos. -3 and pos. -2, respectively, and (iii) three Arabidopsis proteins previously not known to be peroxisome-targeted carry functional PTS1 domains. By contrast, two further predicted PTS1 domains terminating with HKL > and RKM > could not be validated for the Arabidopsis proteins chosen, confirming that the prediction accuracy close to the threshold needs to be further improved by future experimental and computational follow-up studies.

### Computational single-residue permutation analysis of aa residues located upstream of non-canonical PTS1 tripeptides

**Figure 5:**
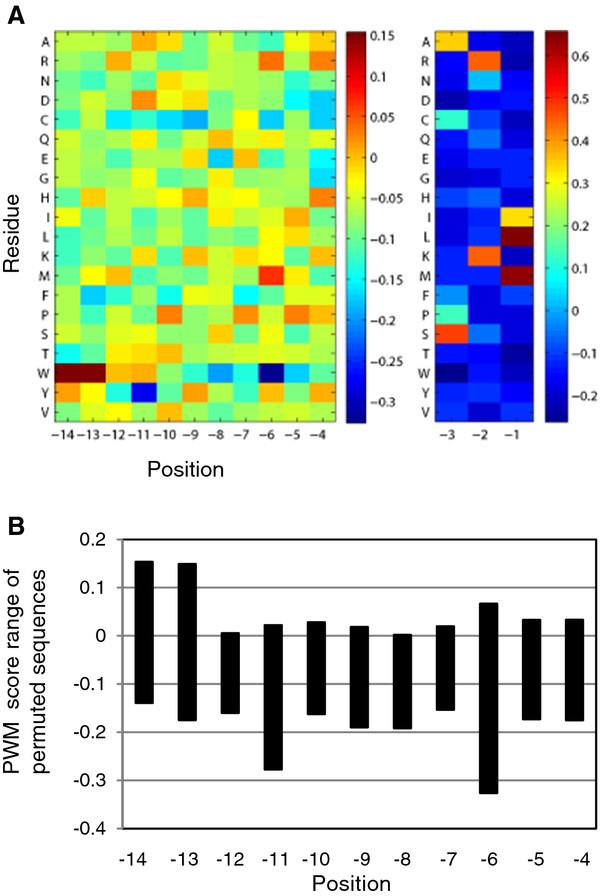
**General PWM score matrix of plant PTS1 proteins displayed as a heat map.** The PWM matrix values are listed in Additional file 1. The values are visualized by a heat map. To account for the different position-specific score ranges, different heat map scales were used for the PTS1 tripeptide and the eleven upstream residues (**A**). From the matrix values of each aa residue the position-specific range of values has been determined (**B**).

PTS1 protein prediction by our PWM models is based on a score matrix in which each of the 20 aa residues at each of the C-terminal 14-aa positions is given a specific prediction score (Figure5A, Additional file 1). Such a position-specific score indicates whether a particular residue at a particular sequence position is predicted to enhance (more positive score, red heat map color) or reduce peroxisome targeting (more negative score, blue). The score matrix shows that, apart from the major role of the C-terminal tripeptide, several upstream residues differ significantly in position-specific abundance between plant PTS1 proteins and non-peroxisomal proteins (Figure5A). These overrepresented residues are predicted to enhance protein targeting to peroxisomes by the PTS1 pathway, particularly in case of non-canonical PTS1 tripeptides [16] (Additional file 1). The total prediction score represents the sum of the 14 position-specific PWM scores for the analyzed sequence of interest.

**Figure 6:**
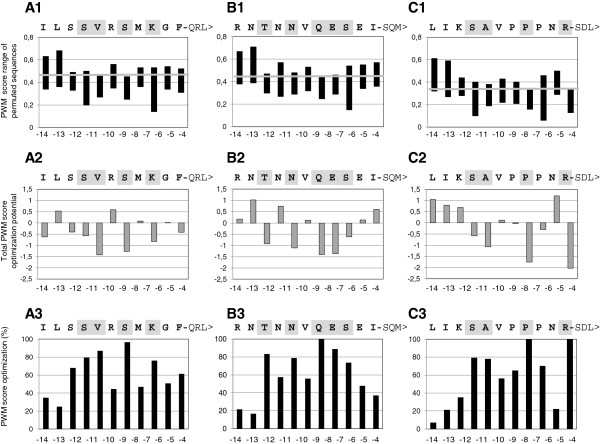
**Computational permutation analysis of three Arabidopsis proteins carrying non-canonical PTS1 domains.** The eleven residues upstream of the novel non-canonical PTS1 tripeptides (pos. -4 to −14) of three Arabidopsis proteins (1, QRL>, At1g18700.2; 2, SQM>, At5g45160.1; 3, SDL>, At5g03730.1) were computationally permuted one by one in all possible 209 combinations (11 x 19 = 209) and the changes in PWM prediction scores investigated. (**A**) Pattern of PWM score range windows of permuted sequences, (**B**) total PWM score optimization potential and (**C**) relative PWM score optimization potential in percentage. The absolute magnitude of the PWM score range window on the y axis and its positioning relative to the PWM prediction score of the original Arabidopsis QRL > sequence (grey line) indicates the absolute optimization potential of a position-specific residue (A). In A1-C1 the PWM prediction score of the original Arabidopsis sequences is indicated by a grey line. Important residues that are predicted to enhance peroxisome targeting are shaded in grey.

To better understand why only some Arabidopsis proteins, among numerous Arabidopsis proteins carrying the same C-terminal tripeptides (e.g., Figure1), were specifically predicted to carry PTS1 domains, we established a new single-residue computational permutation analysis of specific non-canonical Arabidopsis proteins of interest. From the PWM score matrix so-called discriminative features can be derived, which correspond to particular residues at particular positions in a sequence that are associated with a high influence on peroxisome targeting prediction (see [16]). The model thus also allows generating sequences with high targeting probability *de novo* by combining position-specific residues with large positive weights. However, for a particular sequence of interest, e.g. a protein with a non-canonical PTS1 tripeptide, the identification of single residues that possibly enhance or reduce peroxisome targeting using the list of discriminative features is cumbersome. Therefore, we computationally permuted the eleven residues (pos. -4 to −14) upstream of the PTS1 tripeptides in all possible 209 combinations (11 x 19 = 209) in the three Arabidopsis sequences validated as peroxisomal and investigated the effect on the total PWM prediction scores (Figure6).

First, we analyzed the range of PWM prediction score alterations caused by single aa residue point mutations in a position-specific manner and compared this to the PWM prediction score of the original Arabidopsis sequence. The overall position-specific pattern of different PWM score range windows was similar between PTS1 protein sequences, i.e. the magnitude of the window differs per aa position but is constant and sequence-independent at a specific position (Figure5B, Figure6 A1, B1, C1). The reason is that the total PWM prediction scores are calculated by summing up the previously learned position-specific scores for particular residues. For instance, while the three sequences have a relatively small PWM permutation score range window of 0.17 at pos. -7 and −12, the largest window of 0.39 is present at the neighboring pos. -6, where K, S, and P occur in the QRL>, SQM > and SDL > sequence, respectively (Figure6A1-C1). A large window of score distributions indicates that different aa residues have a significant effect on predicted peroxisome targeting. Hence, the algorithms predict that the aa residue at pos. -6 enhances peroxisome targeting to a higher extent than the neighboring residue (pos. -7).

However, these position-specific PWM score windows differ in absolute minimum and maximum values (positioning) for each sequence. For instance, the PWM score range window of pos. -4 of 0.21 in magnitude is located at high values for the QRL > (0.31-0.52) and SQM > sequence (0.36-0.57) but at low values for the SDL > (0.13-0.34) and mostly below the actual prediction value (0.34) of the non-permuted original SDL > sequence from Arabidopsis. The absolute magnitude of the PWM score range window on the y axis and its positioning relative to the PWM prediction score of the original Arabidopsis QRL > sequence (grey line) indicates the absolute optimization potential of a position-specific residue (Figure6A).

To better reveal which upstream positions are predicted to contribute most to peroxisome targeting, we calculated the total PWM score differences of all possible aa point permutations to the original sequence as an indicator of the total optimization potential. In this analysis large negative values indicate that most aa exchanges reduce predicted peroxisome targeting. Hence, these positions have been optimized close to maximum extent by the aa present in the original sequence. By contrast, positive values indicate an “unused” optimization potential, i.e. that several aa exchanges at the given position increase the PWM score for peroxisome targeting. For the QRL > sequence, for instance, K (pos. -6), S (pos. -8), and V (pos. -10) are predicted important residues that enhance peroxisome targeting, while the neighboring residues at uneven positions such as G (pos. -5), M (pos. -7), and R (pos. -9) are predicted sub-optimal residues that do not enhance peroxisome targeting.

To quantify which upstream positions had been most optimized in terms of predicted targeting enhancing elements in the three Arabidopsis proteins of interest, we expressed the PWM prediction score s of a mutated residue r at position s_r,x_ relative to the difference between minimum and maximum score in percentage [(s_max,x_-s_r,x_)/(s_max,x_-s_min,x_) *100]. For the QRL > sequence, for instance, this analysis shows that four residues are close to prediction score maximum (K, pos. -6; S, pos. -8; V, pos. -10; S, pos. -11, grey shaded in Figure 6 A1-3), indicating that these four residues contribute significantly to peroxisome targeting by the weak non-canonical PTS1 tripeptide QRL>. By contrast, R (pos. -9), even though also a basic residue and principally implicated in serving as a targeting enhancing element, is not predicted to be important for peroxisome targeting of the QRL > sequence.

Likewise, for the SQM > sequence, five residues are predicted to contribute most to peroxisome targeting, primarily S (pos. -6), E (pos. -7), and Q (pos. -8) followed by N (pos. -10), and T (pos. -12). For the SDL > sequence, predominantly four residues are predicted to enhance peroxisome targeting, with two optimal residues (100 %, R, pos. -4; P, pos. -7), followed by A (pos. -10) and S (pos. -11). Interestingly, the two proline residues at pos. -6 and −8 are predicted to enhance peroxisome targeting to a considerably lower extent as compared to the neighboring P (pos. -7).

In summary, these permutation analyses of specific single Arabidopsis proteins of interest carrying functional non-canonical PTS1 domains demonstrate that (i) four to five residues positioned between pos. -4 to −12 appear to have been optimized to enable peroxisome targeting, (ii) their exact positioning appears flexible, and (iii) not only basic residues and proline, but also hydroxylated (Ser, Thr), hydrophobic (Ala, Val), and even acidic residues are predicted to be able to enhance peroxisome targeting. Taken together, the experimental and computational data demonstrate that the plant PTS1 motif is more relaxed and that targeting enhancing elements are more diverse and complex than previously assumed. The models allow identification of predicted targeting enhancing and inhibitory elements for specific sequences of interest and their optimization by site-directed mutagenesis.

## Discussion

State-of-the-art prediction algorithms need to address the prediction of non-canonical weak PTS1s. The accuracy of prediction tools is determined by two parameters, high sensitivity and high specificity. The prediction sensitivity in detecting plant PTS1 proteins depends mainly on the ability to identify all functional PTS1 tripeptides of Spermatophyta and, hence, to predict novel “unseen” PTS1 tripeptides that have been absent from training datasets of positive example sequences. Most previously developed prediction tools for fungi and animals were not designed to infer novel PTS1 tripeptides or predict low-abundance proteins because they employed tripeptide-based selection filters [29-32]. By contrast, our newly developed prediction tools for plants could infer novel PTS1 tripeptides, many of which were validated as correct predictions by experimental *in vivo* analyses [16]. By demonstrating in this study that three additional tripeptides are novel non-canonical PTS1 tripeptides, we show that novel tripeptides, even if positioned close to the prediction threshold, are correctly predicted as containing non-canonical PTS1 tripeptides. Thereby, this study increases the number of known plant PTS1s from 51 to 54. With this knowledge more low-abundance plant peroxisomal PTS1 proteins carrying non-canonical PTS1 tripeptides such as QRL>, SQM>, or SDL > can now be identified.

On top of the 32 plant PTS1 tripeptide residues experimentally validated previously [16], the PWM model predicted that ten additional residues might be allowed in plant PTS1 tripeptides ([HKQR][IAVW][QR]>, see Supplemental Dataset 2 online in [16]). One of these residues was validated in the present study, namely Q (pos. -3). Moreover, D (pos. -2) was validated as an allowed PTS1 tripeptide residue, even though the corresponding Arabidopsis sequence was scored slightly below prediction threshold (Table1). Due to the underrepresentation of sequences with non-canonical PTS1 tripeptides in the underlying dataset of positive example sequences, the correct prediction of non-canonical sequences remains challenging, leading to the present inaccuracy that a few false positive (i.e., non-peroxisomal) sequences will be located above prediction threshold (see below) and a few true positive (PTS1 protein) sequences are located below threshold in a prediction grey-zone roughly down to PTS1 protein score position 1100 [16].

The new experimentally verified PTS1 tripeptides add another two residues, Gln (pos. -3) and Asp (pos. -2) to yield in total 34 experimentally validated position-specific residues for the previously reported plant PTS1 motif ([SAPCFVGTLKIQ][RKNMSLH GETFPQCYD][LMIVYF]>), leading to twelve (pos. -3), 16 (pos. -2), and six (pos. -1) allowed aa residues in plant PTS1 tripeptides (Figure4). Hence, the tolerated plant PTS1 motif variation is much higher than previously thought. The former “basic” pos. -2, which was previously considered to be the most conservative aa residue, emerges as the most flexible, with 16 possible residues allowed out of 20 (80 %), even including both acidic residues, Glu and Asp (Figure4). Notably, only specific combinations of the residues of the plant PTS1 tripeptide motif yield functional plant PTS1 tripeptides. All experimentally verified plant PTS1 tripeptides identified to date follow the pattern that at least two high-abundance residues of presumably strong targeting strength ([SA][KR][LMI]>) need to be combined with one low-abundance PTS1 residue to yield functional plant PTS1 tripeptides (x[KR][LMI]>, [SA]y[LMI]>, [SA][KR]z>, Figure4).

In the present study three Arabidopsis proteins that had previously not been associated with peroxisomes were shown to carry functional PTS1 domains. The QRL > decapeptide validated as a functional PTS1 domain derived from the second alternative splice variant of a DNAJ homolog (Figure3, Table1). No DNAJ homolog has been previously shown to be targeted to Arabidopsis peroxisomes. An HSP70 and a DNAJ homolog are reported to be associated with the glyoxysomal membrane in cucumber, and the latter was shown to specifically interact with a cytosolic Hsp70 [33,34]. A watermelon Hsp70 was shown to be dually targeted to both chloroplasts and peroxisomes regulated by alternative translation [35]. The fact that the other three variants of the DNAJ homolog do not carry potential PTS1 domains indicates that the protein is dually targeted to both the cytosol and peroxisomes regulated by alternative splicing. More detailed studies need to address under which conditions this second splice variant is expressed and the full-length protein is targeted to peroxisomes. To date, only a few plant proteins are reported to be dually targeted to peroxisomes and a second cell compartment by alternative splicing. The most prominent example is Arabidopsis transthyretin-like protein, a bifunctional enzyme involved in purine catabolism [17,27,36].

The functional PTS1 domain terminating with the newly identified PTS1, SQM>, belongs to RDH3H2 (At5g45160), a GTP-binding protein and paralog of RDH3 (At3g13870, 67 % sequence identity, 82 % similarity at the aa level, [37], Table1). Loss-of-function mutants of RDH3 are suppressed in epidermal cell file rotation, root skewing, and waving on hard-agar surfaces. RHD3 is involved in the control of vesicle trafficking between the ER and the Golgi compartments [37-40]. Future research needs to address whether the full-length RHDH2 protein is indeed located in peroxisomes.

The functional PTS1 domain terminating with SDL > belongs to the cytosolic Ser/Thr protein kinase CONSTITUTIVE TRIPLE RESPONSE 1 (CTR1, At5g03730), which is an important negative regulator of the ethylene signal transduction pathway regulating plant growth and development [41](for review see [42]). Dark-grown seedlings of “triple response” mutants show an altered response to ethylene. The kinase activity of CTR1 is reported to be regulated by multiple reversible phosphorylation events, leading to significant conformational rearrangements [41]. This mode of post-translational regulation offers the possibility that differential surface exposure of the C-terminal PTS1 domain might cause peroxisome targeting, for instance to transiently eliminate CTR1 from the cytosol.

On the other hand, two predicted non-canonical PTS1 tripeptides could not be validated as functional PTS1 tripeptides for the chosen Arabidopsis sequences, namely those terminating with HKL > and RKM>. The reasons might be several-fold, starting from insufficient sensitivity in detecting weak peroxisome targeting, omission of targeting enhancing elements located upstream of the C-terminal decapeptide in the native protein, to incorrect predictions.

When the full-length cDNA of HSP70T-2 (RKM>) was cloned to the C-terminal end of the reporter protein, the reporter fusion remained cytosolic as well (data not shown). Alternative expression systems including stable Arabidopsis lines might be needed to conclusively investigate whether the two predicted proteins are cytosolic *in vivo*. As a note of caution, PWM predictions of plant proteins with novel non-canonical tripeptides that have not yet been confirmed as functional tripeptides for other sequences should be considered with greater caution compared to predictions of other Arabidopsis proteins carrying confirmed PTS1 tripeptides. Notably, R (pos. -3) was one of the few residues that could also not be confirmed for one positive example sequence [16]. It is important to mention that the PWM prediction algorithms do not consider the similarity of biophysical properties of a residues and deduce predictions solely based on discriminative position-specific aa abundance. Due to the high abundance of SKL > sequences in the underlying dataset and the close codon similarity between Ser (AG[TC]) and Arg (AG[GA]), the two RKL > positive example sequences could have been created by sequencing errors in ESTs and caused the false prediction of RKL > and RKM > sequences as peroxisomal.

Our PWM algorithm combines the C-terminal PTS1 tripeptide and the upstream region (up to 12-aa residues) into a single prediction model. Peroxisome targeting by weak non-canonical PTS1s essentially depends on the presence of targeting enhancing elements in the upstream region. These elements, however, had only been vaguely defined until now. It has been reported for a few sequences that basic residues enhance peroxisome targeting, primarily if located at pos. -4 [26]. Except for the SDL > sequence, none of the other two sequences carried a basic residue directly in front of the non-canonical PTS1, and the SQM > sequence even contained two acidic residues, which are generally very rare in PTS1 domains [14]. It is therefore of interest to identify specific aa residues in a given upstream PTS1 domain that enhance and are essential for peroxisome targeting. To this end, we established in this study a so-called position-specific permutation analysis for non-canonical PTS1 sequences. For each of the newly identified Arabidopsis PTS1 domains carrying novel non-canonical PTS1 tripeptides, we calculated to what extent single aa exchanges in the upstream domain affected the prediction score for peroxisome targeting. In all three sequences, four to five aa residues were identified in the Arabidopsis proteins that represented close-to-optimal residues in term of peroxisome targeting prediction. These data strongly suggest that these residues function as targeting enhancer elements for peroxisome targeting. The exact positioning of these predicted enhancer elements appears relatively flexible between pos. -4 to −12. Most interestingly, not only basic residues and proline, but also hydroxylated (Ser, Thr), hydrophobic (Ala, Val), and even acidic residues are predicted to be able to enhance peroxisome targeting. These predictions are challenging to validate experimentally due to the moderate (SQM>) to low (SDL>) peroxisome targeting efficiency of the original Arabidopsis decapeptides, making it difficult to investigate further reductions semi-quantitatively. Future studies shall address whether such experimental analyses are feasible, for instance, in case of the QRL > sequence.

## Conclusions

Our computational and experimental analyses demonstrate that the plant PTS1 tripeptide motif is more diverse than previously thought and includes many non-canonical sequences. Specific targeting enhancing elements can be predicted for particular sequences of interest and are far more diverse in aa identity and positioning than previously assumed. Machine learning methods become indispensable to predict which proteins, among proteins carrying the same PTS1 tripeptide, contain sufficient enhancer elements for peroxisome targeting.

## Methods

### *In Vivo* subcellular localization studies

For validation of the PTS1 domains that were predicted by the PWM model, the C-terminal 10 residues of Arabidopsis cDNAs were fused to the C terminus of EYFP by PCR using an extended reverse primer (see Additional file 2) and subcloned into the plant expression vector pCAT under control of a double 35 S cauliflower mosaic virus promoter [43] and sequenced. One single aa point mutation occurred in EYFP-7aa-HKL > (At5g50580.1; G(pos. -10)-to-D). For labeling of peroxisomes in double transformants, DsRed-SKL was used [44]. Onion epidermal cells were transformed biolistically as described [19]. The onion slices were placed on wet paper in Petri dishes, stored at room temperature in the dark for approx. 16 h, and analyzed directly or after additional tissue incubation at 10°C for 1 to 6 d.

### Image capture and analysis

Fluorescence image acquisition was performed on a Nikon TE-2000U inverted fluorescence microscope equipped with an Exfo X-cite 120 fluorescence illumination system and either single filters for YFP (exciter HQ500/20, emitter S535/30) and DsRed (exciter D560/40X, emitter D630/60 M). The images were captured using a Hamamatsu Orca ER 1394 cooled CCD camera. Standard image acquisition and analysis was performed using Volocity II software (Improvision) and Photoshop.

### Computational permutation analyses

In order to analyze the influence of single aa point mutations on the peroxisome targeting prediction of a sequence, we generated all possible sequences of length 14 by replacing one single residue at a particular position within the 11-aa upstream region of the PTS1 tripeptide by each of the 19 other aa. For the resulting 11 × 19 = 209 permuted sequences we evaluated the prediction score using the PWM prediction model of size 14 described in [16]. The distribution of position-specific prediction scores was then analyzed with respect to maximum and minimum values and the range between them (considering the score of the original sequence).

The absolute optimization potential of a particular position, i.e. the possibility to enhance peroxisome targeting by a directed residue mutation, is calculated by subtracting the score of the original sequence from that of the maximum position-specific permutation score. The relative optimization potential can then be expressed by dividing the absolute potential by the score range associated with a position. Finally, the total optimization potential associated with a sequence corresponds to the sum of position-specific absolute potentials.

## Competing interests

The authors declare no competing interests.

## Authors’ contributions

GC and ARAK carried out subcloning and *in vivo* subcellular targeting analysis of four and one reporter fusion construct, respectively. TL performed the permutation analysis. SR selected the sequences for experimental analysis, coordinated the project, and wrote the manuscript. All authors read and approved the final manuscript.

## Supplementary Material

Additional file 1 General PWM score matrix. Click here for file

Additional file 2 **Oligonucleotide primers used for cDNA subcloning.** The reverse primers are sorted alphabetically according to the construct name. The XbaI sites in the reverse primers are underlined. One forward primer was used for EYFP amplification and introduced a 5’-NcoI site into the PCR products (5’-AAGTCCATG GTGAGCAAGGGCGAGGA-3’). (DOC 58 kb) Click here for file
