# Supplementary material for: Non-canonical peroxisome targeting signals: identification of novel PTS1 tripeptides and characterization of enhancer elements by computational permutation analysis
Source: BMC Plant Biol. 2012 Aug 11;12:142. doi: 10.1186/1471-2229-12-142 (PMC3487989; doi:10.1186/1471-2229-12-142)
Supplement: Additional file 1 — General PWM score matrix. [file 1471-2229-12-142-S1.doc]

**Additional file 1 – PWM score matrix**

|  | **Position** | |  |  |  |  |  |  |  |  |  |  |  |  |  |
| --- | --- | --- | --- | --- | --- | --- | --- | --- | --- | --- | --- | --- | --- | --- | --- |
| **Res.** | **-14** | **-13** | **-12** | **-11** | **-10** | **-9** | **-8** | **-7** | **-6** | **-5** | **-4** | **-3** | **-2** | **-1** | **Res.** |
| **A** | -0,06 | -0,06 | -0,07 | 0,01 | -0,01 | -0,10 | -0,06 | -0,10 | -0,12 | -0,04 | -0,01 | 0,34 | -0,17 | -0,22 | **A** |
| **R** | -0,08 | -0,09 | 0,01 | -0,06 | -0,10 | -0,10 | -0,06 | -0,07 | 0,03 | -0,06 | 0,03 | -0,16 | 0,46 | -0,23 | **R** |
| **N** | -0,09 | -0,12 | -0,08 | -0,11 | -0,01 | -0,05 | -0,06 | -0,07 | -0,08 | -0,13 | -0,09 | -0,17 | 0,01 | -0,16 | **N** |
| **D** | -0,09 | -0,05 | -0,08 | 0,02 | -0,04 | -0,01 | -0,10 | -0,07 | -0,08 | -0,15 | -0,17 | -0,23 | -0,15 | -0,13 | **D** |
| **C** | -0,12 | -0,06 | -0,16 | -0,13 | -0,16 | -0,19 | -0,09 | -0,03 | -0,17 | -0,08 | -0,18 | 0,12 | -0,10 | -0,22 | **C** |
| **Q** | -0,06 | -0,09 | -0,07 | -0,02 | -0,10 | -0,06 | 0,00 | -0,04 | -0,02 | -0,07 | -0,04 | -0,14 | -0,06 | -0,18 | **Q** |
| **E** | -0,06 | -0,08 | -0,11 | -0,07 | -0,07 | -0,02 | -0,17 | 0,00 | -0,12 | -0,07 | -0,15 | -0,17 | -0,12 | -0,13 | **E** |
| **G** | -0,06 | -0,08 | -0,08 | -0,04 | -0,11 | -0,09 | -0,03 | -0,08 | -0,08 | -0,07 | -0,16 | -0,20 | -0,18 | -0,13 | **G** |
| **H** | -0,10 | -0,01 | -0,06 | -0,05 | -0,03 | 0,01 | -0,04 | -0,03 | -0,07 | -0,08 | 0,03 | -0,09 | -0,07 | -0,18 | **H** |
| **I** | -0,04 | -0,11 | -0,06 | -0,12 | -0,08 | -0,10 | -0,02 | -0,06 | -0,04 | 0,00 | -0,10 | -0,19 | -0,13 | 0,33 | **I** |
| **L** | -0,12 | -0,09 | -0,07 | -0,10 | -0,06 | -0,10 | -0,08 | -0,10 | -0,03 | -0,01 | -0,08 | -0,19 | -0,12 | 0,66 | **L** |
| **K** | -0,12 | -0,08 | -0,10 | -0,02 | -0,12 | 0,00 | -0,05 | -0,01 | -0,03 | -0,11 | 0,00 | -0,12 | 0,44 | -0,21 | **K** |
| **M** | -0,10 | -0,03 | 0,00 | -0,11 | -0,08 | -0,10 | -0,05 | -0,07 | 0,07 | -0,02 | -0,11 | -0,13 | -0,12 | 0,64 | **M** |
| **F** | -0,07 | -0,18 | -0,13 | -0,08 | -0,14 | -0,04 | -0,05 | -0,15 | -0,10 | -0,04 | -0,05 | -0,02 | -0,19 | -0,09 | **F** |
| **P** | -0,07 | -0,12 | -0,05 | -0,10 | 0,03 | -0,08 | -0,07 | 0,02 | -0,05 | 0,03 | 0,00 | 0,13 | -0,18 | -0,19 | **P** |
| **S** | -0,05 | -0,08 | -0,05 | -0,04 | -0,09 | -0,08 | 0,00 | -0,10 | -0,04 | -0,11 | -0,06 | 0,48 | -0,06 | -0,19 | **S** |
| **T** | -0,14 | -0,11 | -0,02 | -0,02 | 0,00 | -0,08 | -0,06 | -0,05 | -0,04 | -0,05 | -0,06 | -0,14 | -0,16 | -0,24 | **T** |
| **W** | 0,15 | 0,15 | 0,00 | 0,01 | -0,09 | -0,12 | -0,19 | -0,14 | -0,33 | -0,17 | -0,10 | -0,26 | -0,15 | -0,21 | **W** |
| **Y** | 0,01 | -0,03 | -0,14 | -0,28 | -0,11 | 0,02 | -0,07 | -0,12 | 0,00 | -0,09 | 0,01 | -0,13 | -0,12 | -0,16 | **Y** |
| **V** | -0,09 | -0,04 | -0,03 | -0,08 | 0,00 | -0,07 | -0,11 | -0,08 | -0,06 | -0,05 | -0,07 | -0,11 | -0,20 | -0,12 | **V** |
|  | **-14** | **-13** | **-12** | **-11** | **-10** | **-9** | **-8** | **-7** | **-6** | **-5** | **-4** | **-3** | **-2** | **-1** |  |
