# Supplementary material for: Non-canonical peroxisome targeting signals: identification of novel PTS1 tripeptides and characterization of enhancer elements by computational permutation analysis
Source: BMC Plant Biol. 2012 Aug 11;12:142. doi: 10.1186/1471-2229-12-142 (PMC3487989; doi:10.1186/1471-2229-12-142)
Supplement: Additional file 2 — Oligonucleotide primers used for cDNA subcloning. The reverse primers are sorted alphabetically according to the construct name. The XbaI sites in the reverse primers are underlined. One forward primer was used for EYFP amplification and introduced a 5’-NcoI site into the PCR products (5’-AAGTCCATG GTGAGCAAGGGCGAGGA-3’). (DOC 58 kb) [file 1471-2229-12-142-S2.doc]

**Additional file 2 – Oligonucleotide primers used for cDNA subcloning**

The reverse primers are sorted alphabetically according to the construct name. The XbaI sites in the reverse primers are underlined. One forward primer was used for EYFP amplification and introduced a 5’-NcoI site into the PCR products (5’-AAGTCCATGGTGAGCAAGGGCGAGGA-3’).

|  |  |  |  |  |
| --- | --- | --- | --- | --- |
| **Construct** | **AGI code** | **Annotation** | **C-terminal 10 aa residues** | **Reverse primer sequence (5’ to 3’)** |
|  |  |  |  |  |
|  |  |  |  |  |
| EYFP-7aa-HKL> | At5g50580.1/2/ At5g50680.1/2 | SUMO-activating enzyme 1B | EDGKGVIEDLS**HKL**> | TATGTCTAGAGTCAAAGCTTGTGGGATAGGTCCTCGATTACACCCTTGTACAGCTCGTCCATGCC |
| EYFP-7aa-QRL> | At1g18700.2 | DNAJ heat shock N-terminal domain-containing protein | ILSSVRSMKGF**QRL**> | TATGTCTAGAGTCATAGCCTCTGGAAACCTTTCATGGACCGCACCTTGTACAGCTCGTCCATGCC |
| EYFP-7aa-RKM> | At2g32120.1/2 | Heat-shock protein 70T-2 | YGATLDLITLQ**RKM**> | TATGTCTAGAGTCACATCTTTCTCTGAAGAGTAATCAAATCAAGCTTGTACAGCTCGTCCATGCC |
| EYFP-7aa-SDL> | At5g03730.1/2 | CONSTITUTIVE TRIPLE RESPONSE 1 | LIKSAVPPPNR**SDL**> | TATGTCTAGAGTCAcaaatccgagcggttgggcggaggaaccgcCTTGTACAGCTCGTCCATGCC |
| EYFP-7aa-SQM> | At5g45160.1 | Root hair defective 3 GTP-binding protein (RHD3) | RNTNNVQESEI**SQM**> | TATGTCTAGAGTCACATTTGTGAGATTTCTGATTCTTGTACATTCTTGTACAGCTCGTCCATGCC |
|  |  |  |  |  |
